# Assessing Long‐Term Neurologic Outcomes in *SAMD9L* ‐Related Ataxia‐Pancytopenia Syndrome

**DOI:** 10.1002/mdc3.14038

**Published:** 2024-04-09

**Authors:** Carla D. Zingariello, Dong‐Hui Chen, Wendy H. Raskind, William B. Slayton, Sub Subramony, Joyce Severance, Megan Feagle, Sonja A. Rasmussen

**Affiliations:** ^1^ Department of Pediatrics University of Florida College of Medicine Gainesville Florida USA; ^2^ Department of Neurology University of Washington Seattle Washington USA; ^3^ Department of Medicine/Medical Genetics University of Washington Seattle Washington USA; ^4^ Department of Neurology University of Florida College of Medicine Gainesville Florida USA; ^5^ UF Health Rehab Center for Kids at Magnolia Parke, University of Florida Gainesville Florida USA; ^6^ Department of Genetic Medicine Johns Hopkins School of Medicine Baltimore Maryland USA

**Keywords:** SAMD9L, ataxia‐pancytopenia syndrome, ataxia, ATXPC

## Abstract

**Background:**

Most published reports on *SAMD9L*‐related ataxia‐pancytopenia syndrome (ATXPC) have emphasized the hematologic findings. Fewer details are known about the progression of neurologic manifestations and methods for monitoring them.

**Cases:**

We present six individuals from two families transmitting a heterozygous variant in *SAMD9L*, exhibiting clinical variations in their hematologic and neurologic findings. Serial motor function testing was used to monitor motor proficiency over a 2 to 3 year period in the proband and his father from Family 1.

**Conclusions:**

Our case series focuses on the neurologic progression in patients with heterozygous variants in *SAMD9L*. Patients with ATXPC should be followed to evaluate a wide range of neurologic manifestations. Serial motor function testing using a standardized method is helpful to track changes in balance and coordination in children and adults with ATXPC and could aid in a future extended natural history study.

## Introduction

The ataxia‐pancytopenia syndrome (ATXPC) was first reported by Li et al^1^ in 1978 in a family with variable levels of ataxia and hematologic cytopenias. In 2016, Chen et al^2^ detected a heterozygous pathogenic variant in *SAMD9L* in another multigenerational family with ATXPC and a different *SAMD9L* variant in the family described by Li, confirming *SAMD9L* as the causative gene. Most published reports on ATXPC focused on familial hematologic disorders, including transient aplastic anemia, myelodysplastic syndrome (MDS) involving monosomy 7, and acute leukemia. More insights into neurologic manifestations and monitoring methods would enhance the understanding of the full clinical spectrum and its pathogenesis.

*SAMD9L* is a tumor suppressor gene located at chromosome 7q21 that inhibits cell proliferation. Gain‐of‐function *SAMD9L* mutations constitutively activate the protein, leading to poor cell division, particularly affecting bone marrow cells. Hematopoietic cells that lose or inactivate the pathogenic variant have a selective advantage. In patients with gain‐of‐function *SAMD9L* mutations, the hematopoietic system can undergo somatic reversion through different mechanisms that result in loss or modification of the pathogenic *SAMD9L* allele. Mechanisms through which somatic reversion occurs include monosomy 7/7q, uniparental disomy (UPD) 7q, and pathogenic inactivating variants that decrease the effects of the germline pathogenic variant.^2^, ^3^, ^4^ Patients with monosomy 7/7q have loss of other genes required for normal hematopoiesis, leading to the development of myelodysplasia. In patients with UPD 7q, the part of chromosome 7 carrying the disease‐causing *SAMD9L* allele is replaced by the wild‐type allele bearing homolog, resulting in cells with two normal copies of chromosome 7.^5^, ^6^ Patients in whom somatic reversion has occurred through UPD or somatic inactivating variants might have normal hematopoiesis occasionally, but remain at risk for development of neurologic manifestations.^7^ These events also explain cases of transient aplastic anemia and disappearance of monosomy 7‐myelodysplastic clones.^8^ Here we report two families with *SAMD9L* variants clinically classified as pathogenic or as a mosaic variant of uncertain significance (VUS) that demonstrate a range of neurologic and hematologic manifestations, and present data on monitoring of ataxia progression using validated methods in two members of family 1.

## Cases

### Family 1

The proband presented for neurologic evaluation at age 10 years because of progressive predominantly cerebellar ataxia. Family history was significant for two brothers with monosomy 7‐MDS. He had normal childhood development. He was found to have mild thrombocytopenia at age 4 years during evaluation as a potential stem cell donor; hematologic work‐up was negative for monosomy 7‐MDS. Based on his prominent ataxia and family history of monosomy 7‐MDS, genetic testing was performed, which revealed a missense VUS in *SAMD9L* (Table 1), present only in 16% of sequencing‐reads, suggestive of mosaicism. The variant was absent from the gnomAD database, and predictive tools indicated the variant likely has a pathologic effect on the protein. At age 11, he was tested on the Bruininks‐Oseretsky Test of Motor Proficiency, 2nd edition (BOT‐2),^9^ and was assessed annually for the period of 3 years (Table 2).

**TABLE 1:** Summary of clinical findings

Case	*SAMD9L* variant, mode of testing	Hematologic findings, age	Neurologic exam findings, age	Neurologic work‐up
Family 1, proband	c.3127C > A, p.Gln1043Lys (VUS), *SAMD9L* single‐gene test (saliva)	CBC: WBCs 7500/mm^3^, hemoglobin 12.3 g/dL, hematocrit 36.3%, platelets 148,000/mm^3^ Bone marrow biopsy: variably cellular marrow, mild hypocellularity for age; maturing multi‐lineage hematopoiesis and mild erythroid hypoplasia, with normal CD20, CD19, and CD10 expression. Cytogenetics: 46 XY; FISH negative for chromosome 7q loss (4 years)	10 years: nystagmus in lateral and upward gaze, MRC grade 4+/5 weakness in neck flexion, finger abduction, hip flexion, ankle dorsiflexion, and toe extension, brisk ankle reflexes with bilateral sustained clonus, upgoing plantar responses bilaterally, wide‐based ataxic gait 11/12 years: previous findings plus overshooting with finger chase test and uncoordinated rapid alternating movements 13/14 years of age: previous findings plus runs with “heavy feet”	Brain MRI (10 years): mild to moderate cerebellar and brainstem atrophy and a few non‐specific FLAIR white matter hyperintensities Brain MRI (12 years): slightly more prominent white matter disease in the bifrontal regions and stable cerebellar atrophy compared to prior Cervical/thoracic MRI (12 years): normal EMG (12 years): moderate, length‐dependent, sensory axonal polyneuropathy
Family 1, proband's father	c.3127C > A, p.Gln1043Lys (VUS), University of Washington (blood)	CBC: WBCs 4700/mm^3^, hemoglobin 13.2 g/dL, hematocrit 38.8%, platelets 147,00/mm^3^ (31 years) CBC: WBCs 3900/mm^3^, hemoglobin 9.2 g/dL, hematocrit 27.8%, platelets 303,000/mm^3^ (41 years)	19 years: slow processing speed, bilateral end gaze and down‐beating nystagmus, symmetric lower extremity hyperreflexia with bilateral ankle clonus, wide‐based gait, inability to tandem gait or stand on either foot 31 years: Previous findings plus overshooting on finger chase, mild dysdiadochokinesia 41 years: Previous findings plus cerebellar dysarthria, positive Romberg	19 years: Brain MRI: bilateral periventricular white matter abnormalities; Normal: cerebrospinal fluid, brainstem auditory and visual evoked potentials, total spine MRI, Brain SPECT, EEG 31 years: Montreal Cognitive Assessment: 19/30; Brain MRI: multiple focal areas of periventricular white matter disease; normal B12, folate; negative HIV, RPR 41 years: EMG: sensory axonal polyneuropathy
Family 1, proband's younger brother	Not tested for *SAMD9L*	CBC: WBCs 2000/mm^3^, hemoglobin 10.7 g/dL, hematocrit 32.5%, platelets 48,000/mm^3^ Bone marrow biopsy: normocellular with multilineage hematopoiesis and 2% blasts with no phenotypic aberrancy. Cytogenetics: monosomy 7 (45, XY, −7), 17/20 metaphases (1 year)	Normal (8 years) Mild nystagmus (9 years)	No brain imaging performed
Family 1, proband's youngest brother	Not tested for *SAMD9L*	CBC: WBCs 4500/mm^3^, hemoglobin 9 g/dL, hematocrit 27.3%, platelets 87,000/mm^3^ Bone marrow biopsy: monosomy 7‐MDS FISH: monosomy 7 in all 20 metaphase cells (6 months)	Nystagmus, difficulty hopping on one foot and going up and down stairs, overshooting with finger chase test (6 years)	Brain MRI (2 years): periventricular and subcortical white matter signal abnormality and mild cerebellar atrophy
Family 2, proband	c.2956 C > T p.Arg986Cys (pathogenic), bone marrow failure gene panel (blood)	CBC: WBCs 5200/mm^3^, hemoglobin 6.9 g/dL, hematocrit 20.4%, platelets 7000/mm^3^, reticulocytes 3.6% (1 year)	Brisk patellar reflexes, upgoing plantar reflexes, overshooting with finger chase, uncoordinated rapid alternating movements, bilateral dysmetria, difficulty with heel‐walking and tandem gait (5 years)	Brain MRI (1 year): mild increased white matter signal about the frontal horns and trigones Brain MRI (5 years): asymmetric enlargement of the atrium of the right lateral ventricular system and mild inferior vermian hypoplasia
Family 2, father	c.2956 C > T p.Arg986Cys single gene familial testing	CBC: reportedly normal	Unknown	Unknown

**TABLE 2:** Ataxia testing for Family 1 proband and proband's father

BOT‐2 measures (years)						
Proband age	Balance	Fine motor precision	Bilateral coordination	Fine motor integration	Manual dexterity	Upper limb coordination
11.6 years	<4	7.1	10.1	15.3	Not performed	Not performed
12.8 years	<4	7.4	8.1	10.1	6.4	8.1
13.9 years	4.25	7.4	6.1	13.3	7.1	16.9
14.6 years	<4	7.8	8.6	10.8	7.8	16.7

SARA scores									
Proband's father age	Gait	Stance	Sitting	Speech disturbance	Finger chase	Nose‐finger test	Fast alternating hand movements	Heel‐shin slide	Total score out of 40
41.7 years	3	2	0	2	1	0	1	1	10
42.4 years	4	4	1	2	3	2	2	2	20
42.8 years	8	6	1	2	2	2	2	2	25
43.7 years	8	6	1	2	2	2	2	2	25

The proband's father, age 44‐years‐old, presented to Neurology at age 19 years for altered gait, dysarthria, and memory loss in the setting of flu‐like symptoms. He had been previously healthy with normal development. Three months later, he developed left lower extremity weakness and coordination difficulties with falling, prompting referral to physical therapy. At age 31 years he presented with gradual decline in balance and increased irritability during a viral illness. He reported confusion, word‐finding difficulties, and trouble performing simple tasks. Ten years later he was seen for progressive difficulty with walking. At neurology visit 6 months later, the Scale for Assessment and Rating of Ataxia (SARA)^10^ was performed and repeated at follow‐ups for two years (Table 2).

**Figure 1:**
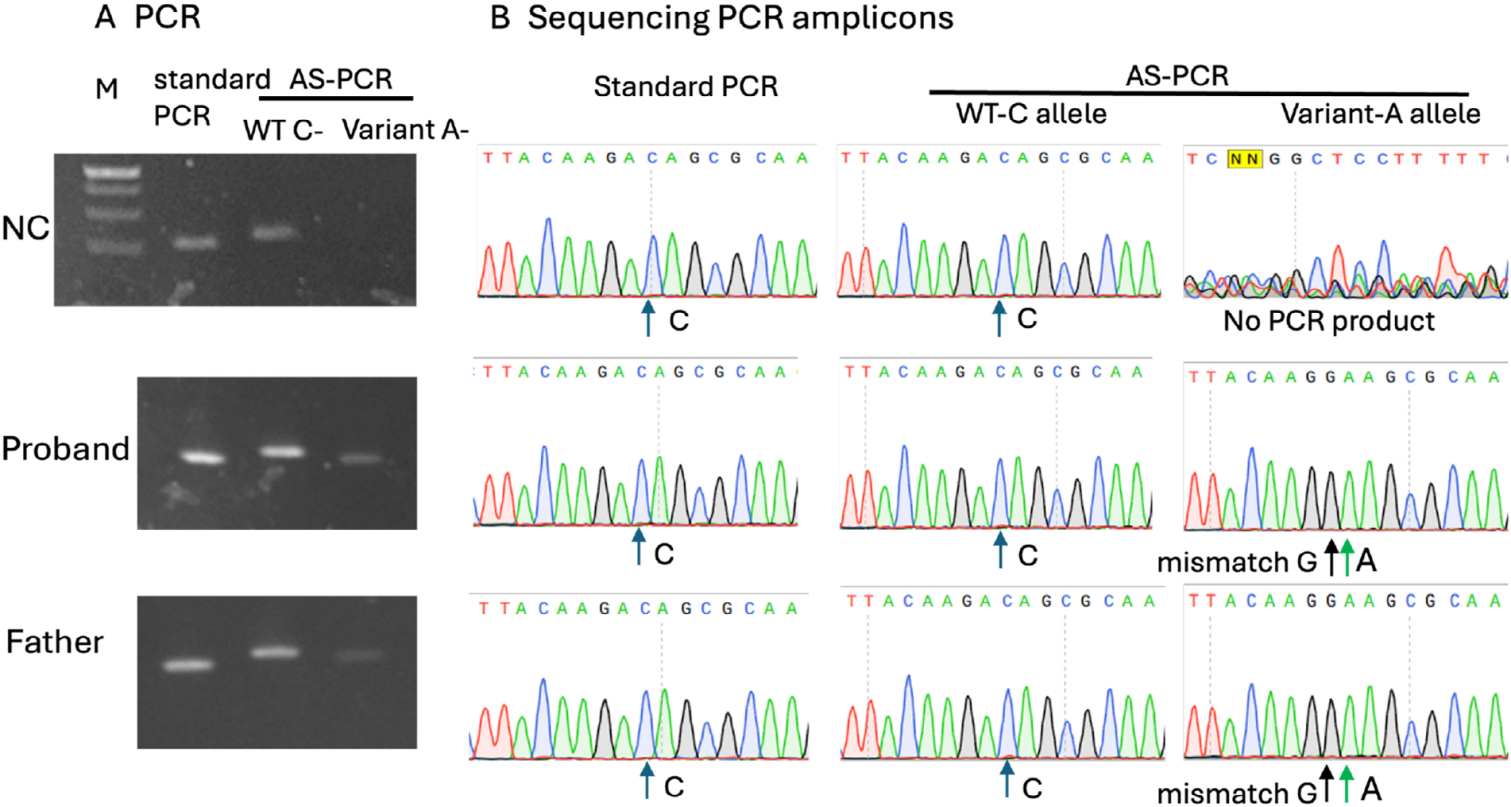
Detecting mosaic *SAMD9L* variant c.3127C > A in Family 1. (**A**) Conventional PCR and allele‐specific PCR specific to wild‐type C allele revealed bands in normal control, proband, and father. Allele‐specific PCR specific to variant A allele amplified products in proband and father but not control. (**B**) Sequencing of PCR amplicons confirmed the presence of variant A allele in proband and father. Amplicons from conventional PCR only showed wild‐type allele in the proband and father, suggesting a very low fraction of variant A in these subjects.

Under an approved IRB protocol at the University of Washington, Sanger sequencing of blood‐derived DNA from the father showed only the wild‐type *SAMD9L* allele. Using allele‐specific PCR primers designed to specifically amplify only the variant or the wild type allele, we detected the *SAMD9L* missense variant seen in his son (Fig. 1), strongly suggesting a low level of mosaicism for cells that retained the germline chromosome 7 bearing the *SAM9DL* variant. Comparative genomic hybridization (CGH) microarray showed loss‐of‐heterozygosity of chromosome 7q, consistent with UPD. The sequencing and CGH results suggested that the majority of blood cells were derived from a stem cell that had undergone somatic reversion.

The older of the proband's two younger brothers developed persistent fevers at age 1 year and was diagnosed with monosomy 7‐MDS (Table 1). An unrelated allogeneic bone marrow transplant (BMT) was performed at age 15 months. At age 8 years, he had no neurologic manifestations. The youngest brother developed fever, rash, and pancytopenia at age 6 months and was diagnosed with monosomy 7‐MDS. He received an unrelated allogeneic HLA‐matched donor BMT at age 7 months. He had normal childhood development and no neurologic complaints but had subtle abnormalities on neurologic exam at age 6 years (Table 1). On the BOT‐2, he scored below average for age in balance, body coordination and fine motor precision, integration, and control. Both brothers received a presumptive diagnosis of ATXPC, given their family history and the monosomy 7 preceding transplant.

### Family 2

The proband presented with pancytopenia and aplastic anemia at age 1 year and had findings of predominantly cerebellar ataxia on neurologic evaluation (age 5 years) and MRI abnormalities (Table 1). He was identified as having a paternally inherited missense variant in *SAM9DL*, previously reported as a pathogenic variant.^5^, ^11^ His father had a normal CBC and was reportedly without neurologic symptoms, although a neurologic exam could not be performed.

## Discussion

Here we present six individuals from two families identified to have a heterozygous variant in the *SAMD9L* gene and various hematologic and neurologic findings. Pathogenic *SAMD9L* variants are associated with a spectrum of clinical severity in both systems, even within a single family, with no evidence of phenotype–genotype correlation.^1^, ^2^, ^3^, ^5^, ^12^ Hematologic abnormalities include pancytopenia, myelodysplasia, often with monosomy 7, or acute myelogenous leukemia. Neurological abnormalities include ataxia (predominantly cerebellar), dysarthria, nystagmus, hyperreflexia, memory and cognitive changes, and white matter changes and cerebellar atrophy on brain MRI.^3^, ^5^, ^12^ As seen in our families, patients can present with a wide variety of hematologic findings, neurologic findings, or both.

Our case series provides greater detail on the neurologic deficits seen in patients with *SAMD9L* variants over time. We also demonstrate the value of following ataxia over a period of years using standardized motor function scales (eg, BOT‐2 or SARA). For the proband in Family 1, the most severe BOT‐2 scores were for balance and remained stable over a period of 3 years. The greatest areas of decline on BOT‐2 were in the composites of fine motor integration and bilateral coordination. Fine motor precision and manual dexterity, although below average at initial testing, remained stable. Improvement in upper limb coordination was seen for unclear reasons. Family 1's proband's father had abrupt worsening of SARA scores following a severe motor vehicle collision with prolonged hospitalization and subsequent loss of independent ambulation. Sitting, finger chase, nose‐finger, fast alternating hand movements, and heel‐shin slide showed only mild worsening over this period, and dysarthria remained stable.

Family 1's proband and his father had EMG evidence of sensory axonal polyneuropathy. Sensory or motor peripheral neuropathy has previously been reported in cases of ATXPC.^6^ The literature suggests that 75% of patients with ATXPC will develop neurologic manifestations and 80% will develop hematologic abnormalities.^3^ Patients with pathogenic *SAMD9L* variants who have not developed or have milder hematologic abnormalities may have had somatic genetic rescue effect in their hematopoietic system through UPD 7q or an inactivating mutation in cis with the aberrant germline *SAMD9L* allele, a phenomenon that appears to occur frequently in patients with *SAMD9L* pathogenic variants.^13^ The time of the somatic reversion occurrence during early development and the repopulation of a tissue could impact revertant clonal clusters size and distribution, which might result in the modification of phenotypic expression in ATXPC cases.^11^ *SAMD9L* is currently included on next‐generation sequencing panels for ataxia and bone marrow failure. However, further genetic testing needs to be considered in patients with findings consistent with ATXPC in whom no *SAMD9L* variants are identified on panel testing, given that somatic genetic reversion can eliminate the *SAMD9L* variant in hematopoietic cells. Evaluation with SNP array, testing using deep target sequencing, and using DNA obtained from other tissues (eg, skin fibroblasts) may be necessary to make a diagnosis.^3^, ^7^

Given the wide presentation of neurologic symptoms seen in patients with *SAMD9L* variants and risk for hematologic abnormalities, patients identified as having pathogenic *SAMD9L* variants should be monitored for both hematologic and neurologic manifestations. Regular testing of coordination and balance using scales such as the BOT‐2 or SARA may be helpful to track patients’ symptoms over time. Ultimately, a comprehensive scale specific to ATXPC incorporating components from the BOT‐2 (in children) or SARA (in adults) would aid in tracking patients over time both individually and collectively as part of a natural history study. This becomes more relevant should prospective therapies emerge, as a disease‐specific scale would be needed to assess objective improvement from any therapeutic intervention.

## Author Roles

(1) Research Project: A. Conception, B. Organization, C. Execution; (2) Statistical Analysis: A. Design, B. Execution, C. Review and Critique; (3) Manuscript Preparation: A. Writing the First Draft, B. Review and Critique.

C.D.Z.: 1A, 1B, 1C, 3A, 3B

D.H.C.: 1B, 1C, 3B

W.H.R.: 1B, 1C, 3B

W.B.S.: 1C, 3B

S.H.S.: 1C, 3B

J.S.: 1C, 3B

M.F.: 1C, 3B

S.A.R.: 1A, 1B, 1C, 3A, 3B

## Disclosures

**Ethical Compliance Statement:** This retrospective study was approved by the University of Florida Institutional Review Board. Written informed patient consent was obtained. We confirm that we have read the Journal's position on issues involved in ethical publication and affirm that this work is consistent with those guidelines.

**Funding Sources and Conflicts of Interest:** This work was supported in part by grants R01NS069719 from the National Institutes of Health and Merit Review Award Number 101 CX001702 from the United States Department of Veterans Affairs. The authors declare that there are no conflicts of interest relevant to this work.

**Financial Disclosures for the Previous 12 Months:** CZ has served on a scientific advisory committee and has industry support from ML Bio. SAR serves on scientific advisory committees for several pregnancy registries, including registries for Wakix (Harmony), Sunosi (Axsome), Nurtec (Biohaven, recently acquired by Pfizer), and the Myfembree (Myovant Sciences). SHS receives funding from the Muscular Dystrophy Association, Wyck Foundation, Friedreich ataxia research alliance and FSHD Society and has industry support from Reata, Retrotope, PTC therapeutics, Avidity Biosciences, Fulcrums, and Vertex. DHC, WHR, WBS, JS, and MF have nothing to disclose.
